# Happy hypoxia in critical COVID‐19 patient: A case report in Tangerang, Indonesia

**DOI:** 10.14814/phy2.14619

**Published:** 2020-10-28

**Authors:** Allen Widysanto, Titis D. Wahyuni, Leonardo H. Simanjuntak, Samuel Sunarso, Sylvia S. Siahaan, Hori Haryanto, Carla O. Pandrya, Ronald C. A. Aritonang, Taufik Sudirman, Natalia M. Christina, Budhi Adhiwidjaja, Catherine Gunawan, Angela Angela

**Affiliations:** ^1^ Siloams Hospital Kelapa Dua Tangerang Indonesia; ^2^ Faculty of Medicine Pelita Harapan University Tangerang Indonesia

**Keywords:** ARDS, COVID‐19, DIC, happy hypoxia, pulmonary intravascular coagulation

## Abstract

Coronavirus Disease 2019 (COVID‐19) is a public health emergency of international concern with increasing cases globally, including in Indonesia. COVID‐19 clinical manifestations ranging from asymptomatic, acute respiratory illness, respiratory failure that necessitate mechanical ventilation and support in an intensive care unit (ICU), to multiple organ dysfunction syndromes. Some patients might present with happy hypoxia, a condition where patients have low oxygen saturations (S_pO2_ < 90%), but are not in significant respiratory distress and often appear clinically well, which is confusing for the doctors and treatment strategies. Most infections are mild in nature and have a relatively low case fatality rate (CFR); however, critical COVID‐19 patients who need support in ICU have high CFR. We would like to report a case of happy hypoxia in a critical COVID‐19‐positive ICU hospitalized patient who survived from Indonesia.

## Graphical abstract

COVID‐19 clinical manifestation ranges from asymptomatic, acute respiratory illness, respiratory failure that necessitate mechanical ventilation and support in an intensive care unit (ICU), to multiple organ dysfunction syndromes. Some patients might be presented with happy hypoxia which is confusing for the doctors and treatment strategies. We would like to report a case of happy hypoxia in a critical COVID‐19‐positive ICU hospitalized patient who survived from Indonesia.

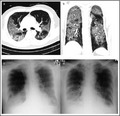

## 1 INTRODUCTION

COVID‐19 is a public health emergency of international concern with millions of confirmed cases globally, including in Indonesia with more than 200,000 confirmed cases to date (Sun et al., 2020). COVID‐19 caused by a droplet‐borne SARS‐CoV‐2 has wide clinical manifestations ranging from asymptomatic, acute respiratory illness, respiratory failure that necessitate mechanical ventilation and support in an intensive care unit (ICU), to multiple organ dysfunction syndromes (MODS; Cascella et al., 2020). While 81% of patients have case fatality rate (CFR) of 2.3%, 5% of the patients develop respiratory failure, septic shock, and MODS resulting CFR of 50% (Siddiqi & Mehra, 2020). Some patients might present with happy hypoxia, a condition where patients have low oxygen saturations as measured by pulse oxymetry (S_pO2_ < 90%), but were not in significant respiratory distress and often appear clinically well, which is confusing for the doctors and treatment strategies (Caputo et al., 2020). Previous studies showed that 9%–11% of COVID‐19 patients needed support in ICU with a CFR of 26%, and only 16% were discharged from the ICU. Among ICU admitted patients, 99% needed respiratory support, including 88% required mechanical ventilation and 11% received non‐invasive ventilation (Grasselli et al., 2020; Remuzzi & Remuzzi, 2020). We report a case of happy hypoxia in a critical COVID‐19‐positive ICU hospitalized patient who survived from Indonesia.

## 2 CASE

**FIGURE 1:**
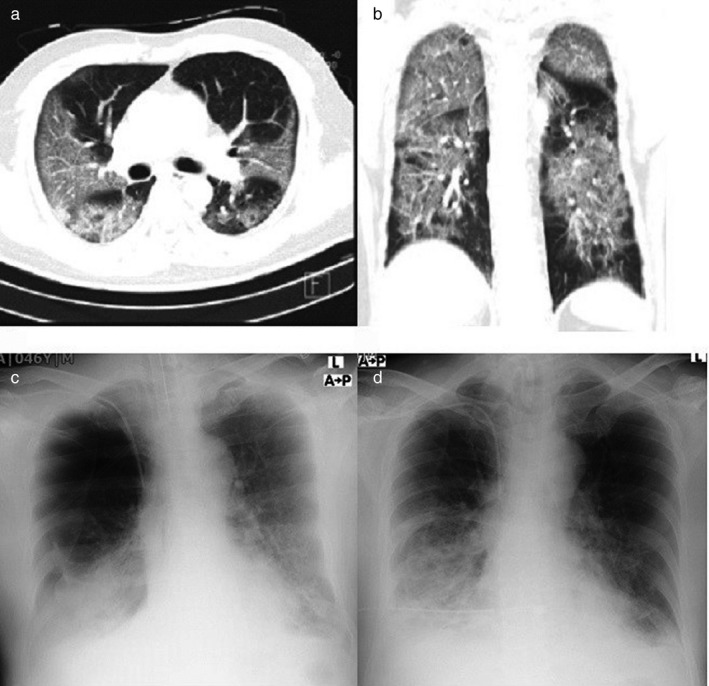
(a b) Coronal and axial slices of chest CT scan on admission showed ground‐glass opacity and multifocal crazy paving patterns involving both lungs, predominantly in peripheral, and paraseptal emphysema on first left lung segment; (c) chest X‐ray on April 10 showed right lung hydropneumothorax and atelectasis, bilateral lung opacities on perihilar and middle to inferior lung fields, cardiothoracic ratio 54% with elongation and calcification of aorta, ETT, and CVP; (d) chest X‐ray post first bronchoscopy, water seal drainage (WSD), and tracheostomy on April 11: Remaining pneumothorax on superior region of right lung, WSD installed with distal tip as high as 10th thoracic vertebra on right lung side

A 48‐year‐old male patient was admitted to our hospital on March 28th, 2020 with presenting symptoms of fever, cough, and dyspnea for 8 days prior to hospitalization. The patient has a history of hypertension on therapy and mild defect of heart septum. The vital signs and physical examinations on admission were as follows: blood pressure 131/78 mmHg, pulse rate 88 times/min, respiratory rate 30 times/min, temperature 37.7°C, S_pO2_ 77% on room air, and rhonchi were found on bilateral lungs. The patient appeared clinically well because he only complained of mild dyspnea, still abled to mobile independently, chatted with doctors, and scrolled on his phone, which is called happy hypoxia condition. Chest CT scan showed ground‐glass opacity (GGO) and multifocal crazy paving pattern involving both lungs, predominantly in peripheral distribution (Figure 1a,b). Chest X‐ray showed bilateral lung opacities on perihilar and middle to inferior lung fields. Laboratory examination showed lymphopenia, increased neutrophil, erythrocyte sedimentation rate, D‐dimer, and C‐reactive protein. Blood gas analysis (BGA) prior to admission showed pH 7.5, P_aO2_ 57 mmHg, P_aCO2_ 29 mmHg, HCO_3_ 22.4 mmoL/L, S_pO2_ 92.2%. His RT‐PCR for COVID‐19 was positive; therefore, he was diagnosed with COVID‐19 with type 1 respiratory failure and acute respiratory distress syndrome (ARDS). The patient was immediately admitted into ICU and given oxygen 15 lpm via a non‐rebreathing mask (NRM) which corrected the S_pO2_ into 95%. Serial BGA showed P_aO2_ 63.5 mmHg which was inadequate; therefore, the NRM was replaced by high flow nasal cannula (HFNC) with F_IO2_ 82%, which corrected the S_pO2_ into 100%. The patient was treated with azithromycin, chloroquine, meropenem, cefoperazone‐sulbactam, kabimidin, tocilizumab, heparin, fluconazole, midazolam, morphine, acetylcysteine, high dose vitamin C, tricyclic H_1_ antagonist, omeprazole, antihypertension drug, furosemide, salbutamol, and bromhexine HCl.

**FIGURE 2:**
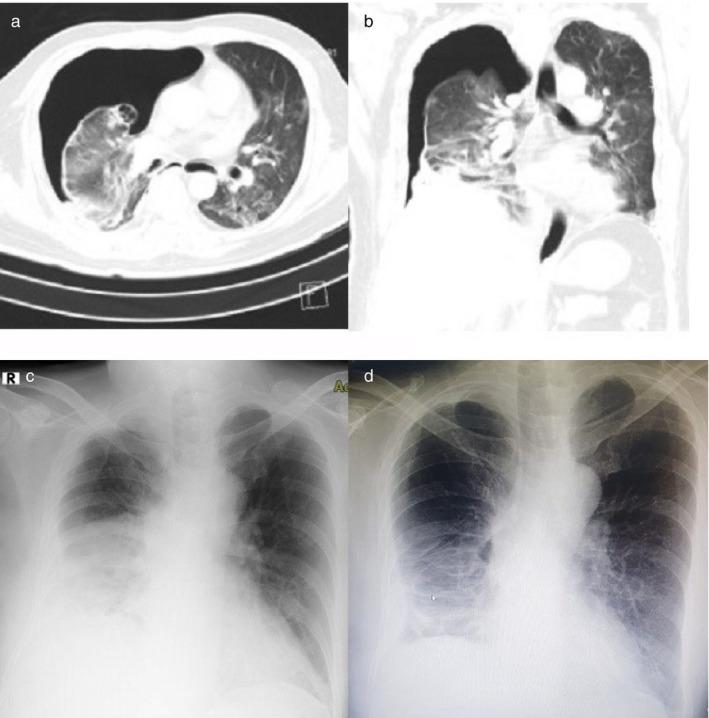
(a, b) Coronal and axial slices of thorax CT scan on April 22 showed ground‐glass opacity with a multifocal fibrotic band with improvement on left lung segment; Pneumothorax on superolateral right lung with right lung collapse; within right lung collapse, was seen ground‐glass opacity, consolidation, thick‐walled cavity with fibrosis, and emphysematous bullae with partially septated with medial and inferior segments of the right lung; minimal pleural effusion on the posterobasal of the right lung; paraseptal emphysema on 1st segment of the left lung. (c) The latest chest X‐ray before hospital discharge showed improvement of right lung pneumothorax and massive right lung fibrosis, and right pleura thickening, (d) chest X‐ray 2 weeks after hospital discharge showed residual but improved massive right lung fibrosis and right pleura thickening

On day 4 of hospitalization (April 1st), the patient developed worsening symptom of dyspnea with S_pO2_ 95% despite HFNC was titrated to F_IO2_ 86%. Serial BGA showed pH 7.45, P_aO2_ 69.9 mmHg, P_aCO2_ 31.9 mmHg, HCO_3_ 22.1 mmoL/L, S_pO2_ 95.5%. Therefore, the patient was intubated and supported with mechanical ventilation. Laboratory examination showed high a D‐dimer count of 4.49; thus, on the next day, intravenous heparin of 250 units/hr was started and continued for 4 days. On day 14 of hospitalization (April 11th), the patient suddenly developed right lung hydropneumothorax and atelectasis as seen on serial thorax X‐ray (Figure 1c); therefore, insertion of water seal drainage (WSD), bronchoscopy, and tracheostomy were done. Bronchoscopy result showed a very thick mucous plug on the right and left main bronchus and *Acinetobacter baumanii* was found in the culture of the bronchial washing. Tracheostomy with mechanical ventilation was set on a pressure control of 15, PEEP 8, and F_IO2_ 65%, and WSD produced 1,000 ml of fluid. The chest X‐ray after the first bronchoscopy as in Figure 1d shows improvement of the hydropneumothorax and atelectasis and serial BGA showed improved oxygenation. On April 9th and 13th, his RT‐PCR for COVID‐19 was negative. On day 18 of hospitalization (April 16th), serial laboratory examination showed overt disseminated intravascular coagulation (DIC) based on the ISTH score (5 without fibrinogen measurement); thus, the patient was given fresh frozen plasma. Seven days after the first bronchoscopy, there were worsening hydropneumothorax and atelectasis seen on chest X‐ray; therefore, second bronchoscopy was carried out with the same results found as before. On day 24 of hospitalization (April 22nd), chest CT scan (Figure 2a,b) showed no significant improvement of the right lung pneumothorax; therefore, WSD was repositioned. On day 36 and 37 of hospitalization (May 4th and 5th), WSD and tracheostomy were removed, respectively. Afterward, the patient was clinically improved, mobilized independently, and S_pO2_ was 98% on room air. The patient was discharged on day 41 of hospitalization (May 9th), and the latest chest X‐ray showed massive right lung fibrosis and right pleura thickening (Figure 2c). During follow‐up on the next 2 weeks, the chest X‐ray showed residual but improved massive right lung fibrosis and right pleura thickening (Figure 2d).

## 3 DISCUSSION

COVID‐19 pneumonia frequently causes hypoxemic respiratory failure attributed to ARDS, impaired hypoxic pulmonary vasoconstriction (HPV), secondary antiphospholipid syndrome, and diffuse pulmonary intravascular coagulopathy (PIC; Archer et al., 2020; McGonagle et al., 2020; Zhang et al., 2020) HPV is the pulmonary circulation's homeostatic response to airway hypoxia in lung diseases, such as pneumonia. HPV constricts pulmonary arteries serving hypoxic lung segments, diverts blood to better ventilated alveoli, and optimizes ventilation/perfusion (V/Q) matching. Impaired HPV explains the profound hypoxia in COVID‐19 pneumonia (Archer et al., 2020). Antiphospholipid antibodies such as anticardiolipin IgA antibodies, anti–β2‐glycoprotein I IgA, and IgG antibodies might present in critically ill patients, including critical COVID‐19 patients. Antiphospholipid antibodies activate endothelial cell and platelets, disrupt natural anticoagulant, and fibrinolytic systems, resulting in a procoagulant state which causes systemic thrombotic events (Willis & Pierangeli, 2011; Zhang et al., 2020). PIC is a lung‐restricted vascular immunopathology associated with COVID‐19, which might evolves into DIC in some pre‐terminal COVID‐19 patients. SARS‐CoV‐2 gains access to the lungs via the ACE2 receptor which is abundantly expressed on type II pneumocytes. Infected pneumocytes cause extensive pulmonary macrophage recruitment and activation, resulting in a clinical picture similar to local macrophage activation syndrome, activation of pulmonary vasculature endothelial cells by IL‐1, IL‐6, and tumor necrosis factor, vasculopathy, immunothrombosis, V/Q mismatch, and refractory ARDS (McGonagle et al., 2020). The authors postulated that this patient initially developed PIC which evolved into DIC resulting in critical condition.

However, the ARDS presentation might be atypical as hypoxemia is profound without appropriate dyspnea, which is defined as silent or happy hypoxia such as in this case (Caputo et al., 2020). Previous study showed only 18.7% COVID‐19 patients reported dyspnea, despite having low P_aO2_: F_IO2_ ratios, abnormal CT scans, and common requirement for supplemental oxygen (Archer et al., 2020). The mechanism of happy hypoxia might be explained by two hypotheses. First, primary “phenotypes” of COVID‐19 pneumonia are divided into type L, characterized by low elastance (i.e., high compliance), low V/Q ratio, low lung weight, low recruitability, and type H, characterized by high elastance, high right‐to‐left shunt, high lung weight, and high recruitability. This patient was initially presented with type L phenotype with relatively well‐preserved lung compliance which explained why he had no significant dyspnea. This also increases minute ventilation which leads to a decrease in P_aCO2_, as found in this patient. The type L patients may remain unchanged for a period and then improve or worsen. In this case, the patient got worse and evolved into type H which resulted in worsened hypoxemia, significant dyspnea, and atelectasis. Second, SARS‐CoV‐2 interferes with mitochondrial O_2_‐sensing and causes mitochondrial‐induced injury, which impairs carotid body function resulting in impaired respiratory drive and reduced dyspnea (Gattinoni et al., 2020). This happy hypoxia condition on admission was the reason we did not immediately intubate and mechanically ventilate this patient, because a previous study showed that doctors should avoid aggressive oxygen therapy to COVID‐19 happy hypoxia such as intubation and mechanical ventilation, because those measures could harm lungs that are inflating on their own (Couzin‐Frankel, 2020; Gattinoni et al., 2020b). NIV was initially given to this patient and then intubation and mechanical ventilation were only started when the patient developed worsening oxygenation.

Markedly raised D‐dimer which is defined as a three to fourfold increase is associated with increased mortality in COVID‐19 infection. This patient had increased D‐dimer on admission and later on developed DIC, which was found in 71.4% of non‐survivors COVID‐19 patients (Tang et al., 2020; Thachil et al., 2020). Also as mentioned before, CFR in COVID‐19 patients with respiratory failure and needed support in ICU were high, up to 66.7% in Indonesia (Grasselli et al., 2020; Remuzzi & Remuzzi, 2020; Siddiqi & Mehra, 2020). This patient was indeed in critical condition due to PIC, DIC, ARDS, respiratory failure, and other complications. However fortunately, the patient survived against the odds with our maximum treatment strategies and recovered, but was left with massive pulmonary fibrosis and pleura thickening.

## CONFLICT OF INTEREST

All authors have nothing to disclose.

## AUTHOR CONTRIBUTION

Dr. Widysanto, Dr. Wahyuni, Dr. Simanjuntak, Dr. Sunarso, Dr. Siahaan, Dr. Haryanto, Dr. Pandrya, Dr. Aritonang, Dr. Sudirman, Dr. Christina and Dr. Adhiwidjaja as a supervisor, consultant, and data collector; Dr. Gunawan has nothing to disclose as a main writer and data collector; Dr. Angela as a main writer and data collector.

## ETHICAL STATEMENT

Ethical clearance from Siloam Kelapa Dua Hospital has been obtained for this manuscript.

## Data Availability

Data available in a public (institutional, general or subject‐specific) repository that issues datasets with DOIs (non‐mandated deposition).
